# A novel selection method of seismic attributes based on gray relational degree and support vector machine

**DOI:** 10.1371/journal.pone.0192407

**Published:** 2018-02-02

**Authors:** Yaping Huang, Haijun Yang, Xuemei Qi, Reza Malekian, Olivia Pfeiffer, Zhixiong Li

**Affiliations:** 1 School of Resources and Geosciences, China University of Mining and Technology, Xuzhou, China; 2 Key Laboratory of Coal Methane and Fire Control, Ministry of Education, China University of Mining and Technology, Xuzhou, China; 3 Institute of China Petroleum Tarim Oilfield Company, Korla, Xinjiang, China; 4 Department of Electrical, Electronic and Computer Engineering, University of Pretoria, Pretoria, South Africa; 5 Department of Mechanical and Industrial Engineering, University of Massachusetts Amherst, Amherst, MA, United States of America; 6 School of Mechatronics Engineering, China University of Mining and Technology, Xuzhou, China; 7 School of Mechanical, Materials, Mechatronic and Biomedical Engineering, University of Wollongong, Wollongong, NSW, Australia; Southwest University, CHINA

## Abstract

The selection of seismic attributes is a key process in reservoir prediction because the prediction accuracy relies on the reliability and credibility of the seismic attributes. However, effective selection method for useful seismic attributes is still a challenge. This paper presents a novel selection method of seismic attributes for reservoir prediction based on the gray relational degree (GRD) and support vector machine (SVM). The proposed method has a two-hierarchical structure. In the first hierarchy, the primary selection of seismic attributes is achieved by calculating the GRD between seismic attributes and reservoir parameters, and the GRD between the seismic attributes. The principle of the primary selection is that these seismic attributes with higher GRD to the reservoir parameters will have smaller GRD between themselves as compared to those with lower GRD to the reservoir parameters. Then the SVM is employed in the second hierarchy to perform an interactive error verification using training samples for the purpose of determining the final seismic attributes. A real-world case study was conducted to evaluate the proposed GRD-SVM method. Reliable seismic attributes were selected to predict the coalbed methane (CBM) content in southern Qinshui basin, China. In the analysis, the instantaneous amplitude, instantaneous bandwidth, instantaneous frequency, and minimum negative curvature were selected, and the predicted CBM content was fundamentally consistent with the measured CBM content. This real-world case study demonstrates that the proposed method is able to effectively select seismic attributes, and improve the prediction accuracy. Thus, the proposed GRD-SVM method can be used for the selection of seismic attributes in practice.

## Introduction

Seismic attributes have been used an important feature for the purpose of reservoir prediction to recognize reservoir patterns [1–4]. However, the relationships between seismic attributes and reservoir lithology, fluid properties, and other parameters are rather complex. Several types of seismic attributes can even cause various adverse effects for reservoir prediction. For example, the instantaneous phase and frequency attributes are important information in seismic exploration, but these seismic attributes cannot show the stratigraphic boundary clearly [5]. The coherence attribute can increase the geological information on the time slice, but it damages the information on the vertical section at the same time [6]. Kalkomey found that the introduction of irrelevant attributes for reservoir prediction can lead to false predictions [7]. Barnes suggested that redundant seismic attributes in the candidate pool may confuse seismic interpretation [8]. Redundant seismic attributes also occupy a large storage space and require a high computational time [9]. In addition, the interaction and inner relationship between different seismic attributes in a large attributes space may lead to repetition and waste of resources. Hence, it is imperative to select the most useful/reliable seismic attributes to perform precise and efficient reservoir prediction [10–12].

In order for the selection of seismic attributes, Hampson et al. proposed the stepwise regression method [13]. Dorrington et al. presented the selection method of seismic attribute by using a genetic algorithm, which can select the optimal number and type of seismic attributes for the prediction of porosity [10]. Gao et al. presented a novel selection method called constrained main component analysis [14]. Ahmed et al. introduced the abductive networks to predict reservoir properties from seismic attributes. The abductive networks simultaneously selected the most relevant attributes and constructed an optimal nonlinear predictor [12]. Zhang et al. proposed a selection method of seismic attributes based on SVM, which selected attributes from various types of seismic attributes [15]. Iturrarán-Viveros used the Gamma test to analyze the data, selected the combination of seismic attributes that have the smaller Gamma statistic and trained the Artificial Neural Network (ANN) to learn to estimate porosity [16]. Qi et al. selected nine attributes that have high coefficients with the measured coalbed methane (CBM) content value, and then performed correlation analysis among these nine attributes and selected four seismic attributes with the smallest correlation coefficients to identify the CBM-enriched areas [17]. Wang et al. combined rough sets and K-L transform approach to select seismic attributes [4]. Galvis et al. presented a methodology that uses pattern recognition to select the best seismic attributes [18]. Gholamiet al. used a committee model with bat-inspired optimization algorithm to estimate porosity based on seismic attributes [19]. These excellent researches have addressed the problem of seismic attribute selection by providing the solutions from different viewpoint. However, to the best of our knowledge, the relationships between the seismic attributes and between the attributes and reservoir parameters have not been exploited in the existing selection process yet. It is worth investigating these relationships in the attribute selection because existing publications have indicated close connections between attributes and reservoir parameters [13–19] and can be discovered by advanced signal processing methods [20–22].

In order to select the most useful seismic attributes, this paper presents a novel selection method by addressing the relationships between the seismic attributes and reservoir parameters. A two-hierarchical structure was established based on the integration of gray relational degree (GRD) and support vector machine (SVM). A real world case study in southern Qinshui basin, China, has been carried out to examine the performance of the proposed GRD-SVM method. The analysis results show satisfactory prediction accuracy on the CBM content.

The rest of this paper is organized as follows. Firstly, the basic principles of GRD and SVM are elaborated, and then the basic idea of selection of seismic attributes based on GRD-SVM is introduced. Thirdly, the case study using the real world data is carried out to illustrate the effectiveness of the proposed method. Lastly, the conclusions are drawn.

## Methods

### Gray relational degree (GRD)

GRD was pioneered by Deng in 1984 [23]. GRD provides a simple scheme to analyze the relationships of time series, even the information provided is little informative about the objective. GRD is an analysis tool in essence, which is based on the distance between reference and comparative positions by its geometry characteristics. For a discrete time series, if the increment of two discrete time series is equal or close to equal in one period of time, the GRD of the two series is high; otherwise, the GRD is low. The basic calculation steps of GRD are as follows:

For a time interval [*a*,*b*], *b*>*a*≥0, make Δ*t*_*k*_ = *t*_*k*_−*t*_*k*−1_, *k* = 2,3,…,*n*, $[a,b]=\overset{n}{\underset{k=2}{\cup }}\Delta t_{k}$ $\Delta t_{k}\cap \Delta t_{k-1}=\emptyset$, *k* = 2,3,…,*n*. The two discrete time series in the time interval [*a*,*b*] are *X*_1_ = (*x*_1_(*t*_1_),*x*_1_(*t*_2_),⋯,*x*_1_(*t*_*n*_)), *X*_2_ = (*x*_2_(*t*_1_),*x*_2_(*t*_2_),⋯,*x*_2_(*t*_*n*_)).

Then, *y*_*i*_(*t*_*k*_) = *x*_*i*_(*t*_*k*_)−*x*_*i*_(*t*_*k*−1_), (*i* = 1,2;*k* = 2,3,…,*n*) represents the increment of the time series in time *t*_*k*−1_ to *t*_*k*_.

$D_{i}=\frac{\sum_{k=2}^{n}\left|y_{i}(t_{k})\right|}{n-1}(i=1,2)$ represents the increment average value of the absolute value in each period of the time series. $z_{i}(t_{k})=\frac{y_{i}(t_{k})}{D_{i}}(i=1,2,k=2,3,\ldots ,n)$ represents the equalization value of increment in time *t*_*k*−1_ to *t*_*k*_ of the time series. The gray correlation coefficient *ξ* of time series *X*_1_ and *X*_2_ in time *t*_*k*−1_ to *t*_*k*_ is provided below:

$$\xi (t_{k})=\left\{ \begin{array}{l} \mathrm{sgn}(z_{1}(t_{k}).z_{2}(t_{k}))\frac{1}{1+\frac{1}{2}\|z_{1}(t_{k})|-|z_{2}(t_{k})\|+\frac{1}{2}(1-\frac{\min(|z_{1}(t_{k})|,|z_{2}(t_{k})|)}{\max(|z_{1}(t_{k})|,|z_{2}(t_{k})|)})};z_{1}(t_{k})\cup z_{2}(t_{k})\neq 0\\ 1\;;z_{1}(t_{k})\cap z_{2}(t_{k})=0 \end{array}\right.$$ (1)

Where *k* is a serial number of time. Eq (1) is used to calculate the gray correlation coefficient of time series *X*_1_ and *X*_2_ in time *t*_*k*−1_ to *t*_*k*_. sgn(*z*_1_(*t*_*k*_).*z*_2_(*t*_*k*_)) is a symbol function, which reflects the correlations of the two sequences. In addition, when *z*_1_(*t*_*k*_).*z*_2_(*t*_*k*_)>0, then *ξ*(*t*_*k*_)>0, and when *z*_1_(*t*_*k*_).*z*_2_(*t*_*k*_)<0, then *ξ*(*t*_*k*_)<0.‖*z*_1_(*t*_*k*_)|−|*z*_2_(*t*_*k*_)‖ is the increment of the constitute difference, and $\left(1-\frac{\min(|z_{1}(t_{k})|,|z_{2}(t_{k})|)}{\max(|z_{1}(t_{k})|,|z_{2}(t_{k})|)}\right)$ is the constituent ratio. When the increment between the two time series in time *t*_*k*-1_ to *t*_*k*_ is equal or close to equal, ‖*z*_1_(*t*_*k*_)|−|*z*_2_(*t*_*k*_)‖ and $\left(1-\frac{\min(|z_{1}(t_{k})|,|z_{2}(t_{k})|)}{\max(|z_{1}(t_{k})|,|z_{2}(t_{k})|)}\right)$ are close to zero. At this time, the gray correlation coefficient of the two time series in time *t*_*k*−1_ to *t*_*k*_ is high (close to 1); otherwise, the gray correlation coefficient is low.

After we calculate the gray correlation coefficient, the GRD in time interval [*a*,*b*] can be calculated by the Eq (2).

$$r=\frac{1}{b-a}\sum_{k=2}^{n}\Delta t_{k}.\xi (t_{k})$$ (2)

Where *r* is the GRD of the time series *X*_1_ and *X*_2_.

When we assume *t*_*k*_ = *k*, *k* = 1,2,…,*n*, Eq (2) can be transformed to the following constrained formation:

$$r=\frac{1}{n-1}\sum_{k=2}^{n}\xi (k)$$ (3)

### Support vector machine (SVM)

SVM, which is a set of related supervised learning methods for classification and regression, was first introduced by Vapnik [24]. SVM provides the best generalization accuracy classifier by maximizing the margin between two classes in a feature space. Because of this, it has been widely applied in several fields such as classification, regression, face recognition, and time series prediction. Support vector regression is an application of SVM for function regression. The basic theory of SVM has been detailed described in several literatures, and thus, the description of the theory will not be repeated in this paper. We consider linear support vector regression machine as an example and briefly introduce the basic principle of SVM.

In a given training sample set {(*x*_*i*_,*y*_*i*_),*i* = 1,2…,*l*}, *x*_*i*_ ∈ *R*^*n*^ represents the input value, *y*_*i*_ ∈ *R* represents the corresponding target value, *l* is the number of samples. *f*(*x*) = <*w*,*x*>+*b* is used for fitting the sample data set {*x*_*i*_,*y*_*i*_}, and *b* is the offset value.

$${\left|y-f\left(x\right)\right|}_{\varepsilon }=\{\begin{array}{c} 0,if\left|y-f\left(x\right)\right|\leq \varepsilon \\ \left|y-f\left(x\right)\right|-\varepsilon ,if\left|y-f\left(x\right)\right|>\varepsilon \end{array}$$ (4)

In Eq (4), *ε* is the insensitive loss function, which is directly related to function estimation precision, and its value is greater than zero. The main purpose of this study is to construct a function *f*(*x*), and, at the same time, ensure that the distance between the function and target is less than *ε*. Thus, for those unknown samples *x*, the function *f*(*x*) can optimally estimate the corresponding target value.

In the linear case, we can assume *f*(*x*) for the following form:

f(x)=<w⋅x>+b (5)

Where *w* ∈ *R*^*n*^ is the weight vector of function *f*(*x*). *x* ∈ *R*^*n*^ is the input value, <.> is the inner product, and *b* ∈ *R* is the offset value of function *f*(*x*).

For a given training sample, (*x*_*i*_,*y*_*i*_), *i* = 1,2,…,*l*, *x* ∈ *R*^*n*^, *y* ∈ *R*, the optimization problem is as follows:

$$\min\frac{1}{2}{\left\|w\right\|}^{2}$$ (6)

Subject to: $\begin{array}{l} y_{i}-<w\cdot x_{i}>-b\leq \varepsilon ,\\ or<w\cdot x_{i}>+b-y_{i}\leq \varepsilon ,i=1,\ldots ,l \end{array}$

When a given constraint condition is not able to solve Eq (6), the slack variables *ξ*_*i*_ and $\xi_{i}^{*}$ are introduced, which represent the distance from the actual values to the corresponding boundary values of *ε* (the insensitive loss function). Eq (6) can be transformed into the following constrained formation:

$$\underset{w,b,\xi }{\min}\frac{1}{2}{\left\|w\right\|}^{2}+C\sum_{i=1}^{l}\left(\xi_{i}+\xi_{i}^{*}\right)$$ (7)

Subject to: $\begin{array}{l} <w\cdot x_{i}>+b-y_{i}\leq \varepsilon +\xi_{i}\\ or\begin{array}{cc} & y_{i}-<w\cdot x_{i}>-b \end{array}\leq \varepsilon +\xi_{i}^{*}\\ \xi_{i},\xi_{i}^{*}\geq 0,i=1,2,\cdots ,l \end{array}$

Where *C* is the penalty coefficient, which is taken as the regularized constant that determines the trade-off between the empirical error (risk) and regularization term. Eq (7) represents quadratic programming problems with linear inequality constraints, which can be solved using the Lagrange multiplier method, i.e.,

$$f(x)=<w\cdot x>+b=\sum_{i=1}^{l}\left(a_{i}^{}-a_{i}^{*}\right)<x_{i}\cdot x>+b$$ (8)

Where *a*_*i*_ and $a_{i}^{*}$ are called Lagrange multipliers.

### Selection method of seismic attributes based on GRD and SVM

**Fig 1:**
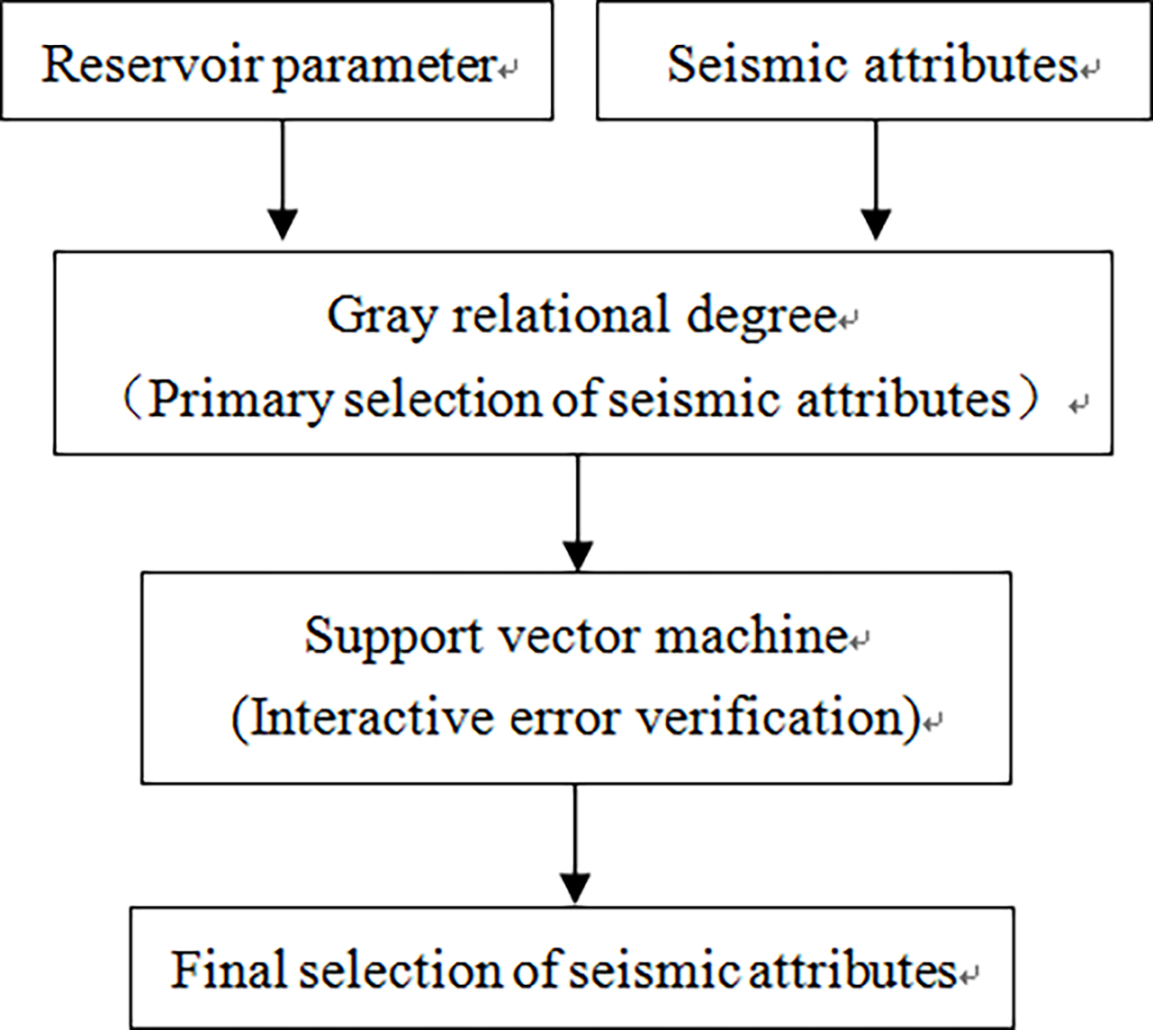
The flow chart of seismic attributes selection method.

GRD can truly reflect the degree of closeness of relative changes in the time series. The GRD between seismic attributes and reservoir parameters, and the GRD between different seismic attributes can be calculated to achieve primary selection of seismic attributes. SVM is very suitable for small samples, nonlinear cases, and other issues. It has better generalization and promotion capabilities. Thus, SVM can be used to perform interactive error verification of known samples and achieve final selection of seismic attributes. The flow chart of the selection method of seismic attributes based on GRD and SVM is shown in Fig 1.

The basic idea of GRD-SVM is as follows:

1. GRD is used to select seismic attributes for preprocessing, i.e., based on the GRD between seismic attributes and reservoir parameters, 6–8 types of seismic attributes are initially selected, which have high correlation with reservoir parameters, in order to effectively remove redundant attributes and reduce the number of seismic attributes used for reservoir prediction.
2. The 6–8 types of seismic attributes that were selected may have high GRD with each other. The GRD between the selected seismic attributes is calculated, and seismic attributes with higher GRDs are clustered into one category. The seismic attribute that has the largest GRD with reservoir parameters is selected from each category; thus, 3–5 types of sensitive seismic attributes are selected.
3. SVM is used to perform interactive error verification for known samples. When a seismic attribute is selected, a particular sample is excluded from testing. The rest of the samples in the training set are used to calculate the predicted root mean square error (RMSE). The above procedure is repeated for all the samples. The calculated RMSEs of all the samples are added and then divided by the total number of samples to obtain the RMSE of one seismic attribute. The number of seismic attributes is increased, and the above steps are repeated. The RMSE of different types of seismic attributes are calculated, and seismic attributes with the smallest RMSE are selected.

## Case study

CBM is one type of gas spontaneously occurring in coal seams. The prediction of CBM content is a crucial factor for production safety in coal mines and formulation of plans for the development of CBM resources. The use of seismic attributes to predict CBM content has made great progress [25, 26], but there is a lack of research on selection methods of seismic attributes for the prediction of CBM content.

The research area was located at the southern part of the Qinshui Basin. The coal series was developed mainly in the Lower Permian Shanxi group and the Upper Carboniferous Taiyuan group. A total of 15 coal beds were found in these strata with a mean total thickness 136.02 m. Three coal seams in the Shanxi Group and fifteen coal seams in the Taiyuan Group are minable. The thickness of the three coal seams in the Shanxi Group ranges from 6.49 to 7.45 m with an average thickness of 6.79 m. The main structure is typical of synclinal and anticlinal composite folds extending to NNE and NE, respectively [17].

Qi and Zhang [17] studied the seismic attributes of this area. A total of 64 seismic attributes, including coal seam reflected waves, amplitude, complex seismic trace, and sequence statistics attributes were used. Table 1 is the seismic attributes used in this study, the calculation method of each seismic attribute can see the help manual of Landmark and Geomodeling. The selected seismic attributes were combined based on the Dempster-Shafer evidence theory, and CBM enriched areas were predicted. In order to verify the selection method of seismic attributes proposed in this paper, the seismic attributes of Qi's literature were used to select and analyze.

**Table 1:** Seismic attributes used in this study. Code was used to refer to the name in figures.

Code	Attribute name	Code	Attribute name
1	Root Mean Square Amplitude	33	Number of troughs
2	Average absolute amplitude	34	Covariance coefficient to next CDP
3	Maximum peak amplitude	35	Correlation window time shift to next CDP
4	Average peak amplitude	36	Average signal-to-noise ratio
5	Maximum Trough amplitude	37	Correlation length
6	Average Trough amplitude	38	Correlation components
7	Maximum absolute amplitude	39	Karhunen-loeve signal complexity
8	Total absolute amplitude	40	Instantaneous amplitude
9	Total amplitude	41	Instantaneous frequency
10	Average energy	42	Instantaneous phase
11	Total energy	43	Average curvature
12	Mean amplitude	44	Gaussian curvature
13	Variance in Amplitude	45	Maximum curvature
14	Skew in amplitude	46	Minimum curvature
15	Kurtosis in amplitude	47	Maximum positive curvature
16	Average reflection strength	48	Minimum negative curvature
17	Average instantaneous frequency	49	Effective bandwidth
18	Average instantaneous phase	50	Dip
19	Slope of reflection strength	51	instantaneous main frequency
20	Slope of instantaneous frequency	52	Dip-Azimuth
21	Instantaneous bandwidth	53	Strike
22	Arc length	54	Lambertian reflectance
23	Average zero crossings frequency	55	Local variability
24	Dominant frequency series	56	Second-Order derivative
25	Peak spectral frequency	57	Semblance
26	Spectral slope from peak to maximum frequency	58	Relative acoustic impedance
27	Percent of greater than threshold	59	Sweetness
28	Percent less than threshold	60	Cosine of instantaneous phase
29	Energy half-time	61	Instantaneous acceleration
30	Slope at energy half-time	62	Thin bed indicator
31	Ration of positive to negative sample	63	Response phase
32	Number of peaks	64	Absorption attribute

The measured CBM content of the seven wells located in 3# coal seam of Qinshui Basin were 18.02m^3^/t, 17.58m^3^/t, 16.86m^3^/t, 12.51m^3^/t, 10.12m^3^/t, 9.79m^3^/t and 8.68m^3^/t, respectively. Based on the value of GRD between the seismic attributes and CBM content, we selected instantaneous amplitude, instantaneous bandwidth, instantaneous main frequency, instantaneous frequency, minimum negative curvature, instantaneous phase, maximum positive curvature, and absorption attribute. Then we calculated the GRDs between the eight types of seismic attributes, which were shown in Table 2.

**Table 2:** GRDs of the eight types seismic attributes.

Code	40	21	51	41	48	42	47	64
40	1	0.0139	-0.5797	-0.1878	-0.0047	0.1083	-0.0309	0.2426
21		1	-0.1792	-0.5641	-0.0840	-0.2102	0.7049	0.3401
51			1	0.1259	0.2657	-0.2907	-0.1659	-0.3578
41				1	-0.3859	-0.4001	0.5407	0.1373
48					1	0.0236	-0.2894	-0.0234
42						1	-0.1515	0.0687
47							1	0.3432
64								1

As shown in Table 2, some seismic attributes have high GRD with other seismic attributes. The GRD between instantaneous bandwidth and maximum positive curvature is 0.7049, and the GRD between instantaneous amplitude and instantaneous main frequency is -0.5797. Based on the selection principle of seismic attributes with a greater GRD, we further selected instantaneous amplitude, instantaneous bandwidth, instantaneous frequency, minimum negative curvature, instantaneous phase, and absorption attribute as sensitive seismic attributes. SVM was used to perform interactive error verification between the sensitive seismic attributes and the known CBM content, and the final selection of seismic attributes was achieved based on the RMSE between the predicted and actual values. The specific process is as follows:

1. When a particular type of seismic attribute was selected, the data of one well was removed from the training data set (this well is usually known as the blind well). The SVM training data of the other six wells were used, and the obtained training results were used to predict the result of the blind well. The RMSE between the predicted and the theoretical values was calculated.
2. This process was sequentially performed for all the seven wells present in this work area. The RMSE of the seismic attribute was added and then divided by 7 to obtain the RMSE of this seismic attribute.
3. The types of seismic attributes were increased, and the above process was repeated to obtain the RMSE of different types of seismic attributes, as shown in Table 3.

**Table 3:** Number of seismic attributes and the corresponding RMSE.

Number of seismic attributes	1	2	3	4	5	6
RMSE	1.2092	1.5110	0.6731	0.6305	0.6479	0.7611

**Fig 2:**
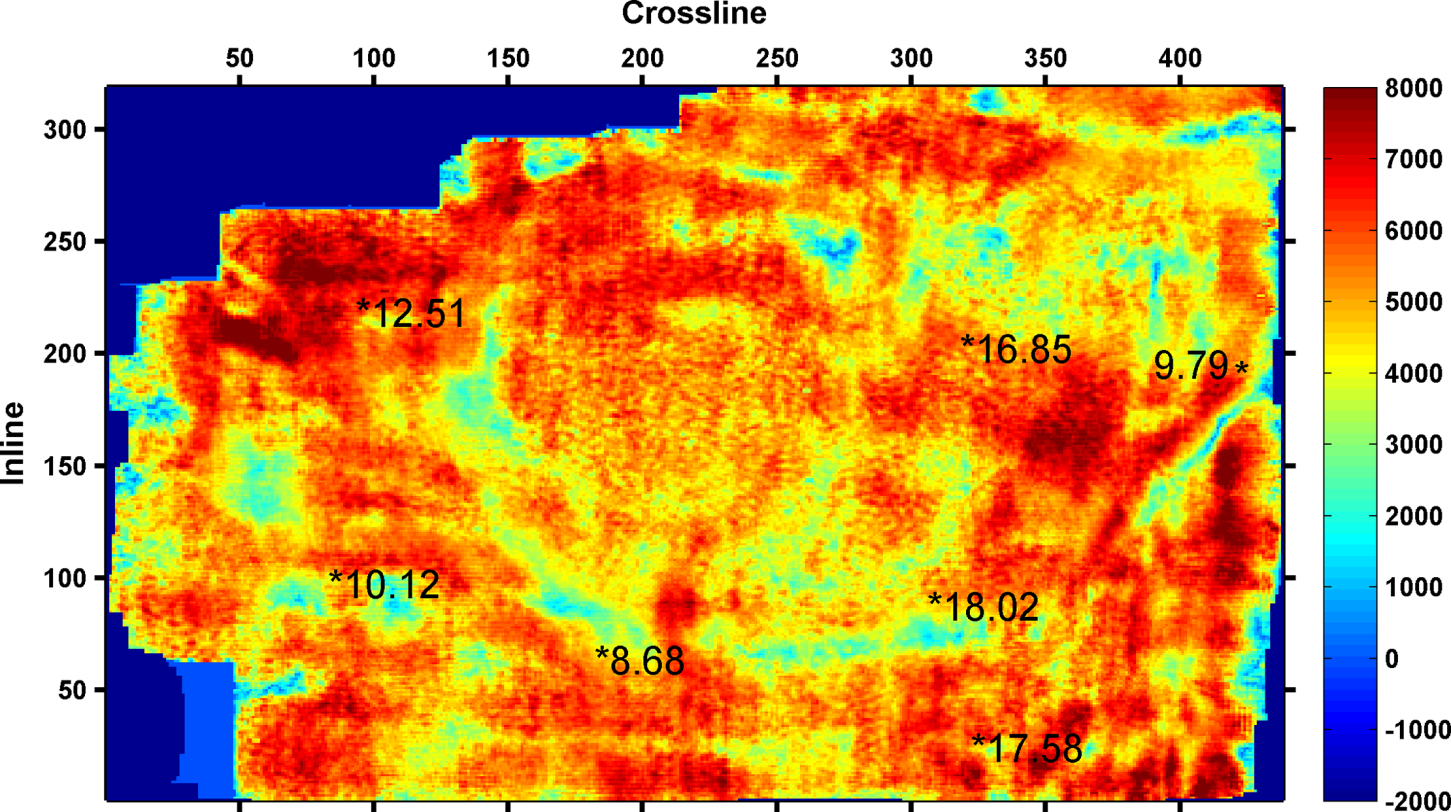
Instantaneous amplitude attribute.

**Fig 3:**
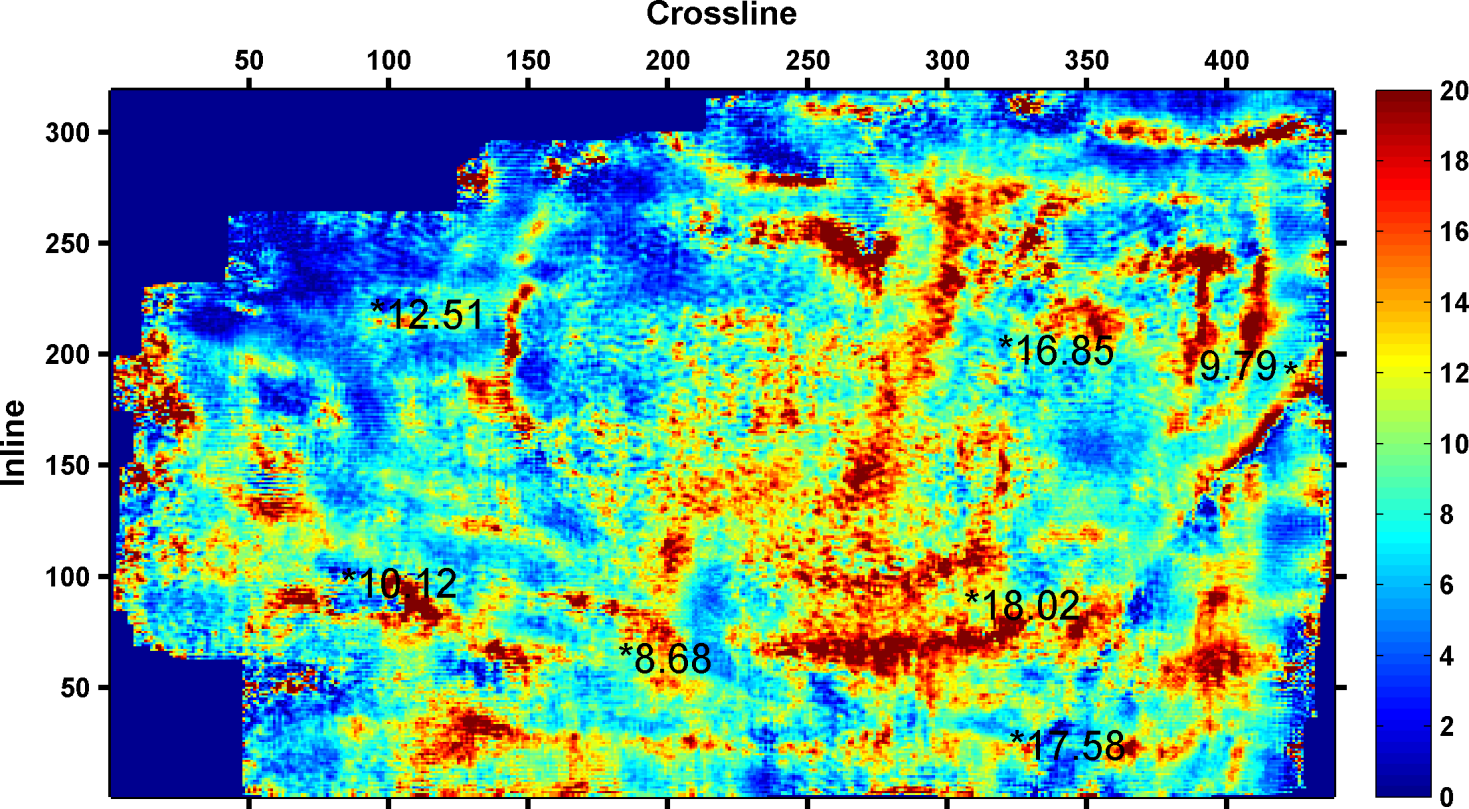
Instantaneous bandwidth attribute.

**Fig 4:**
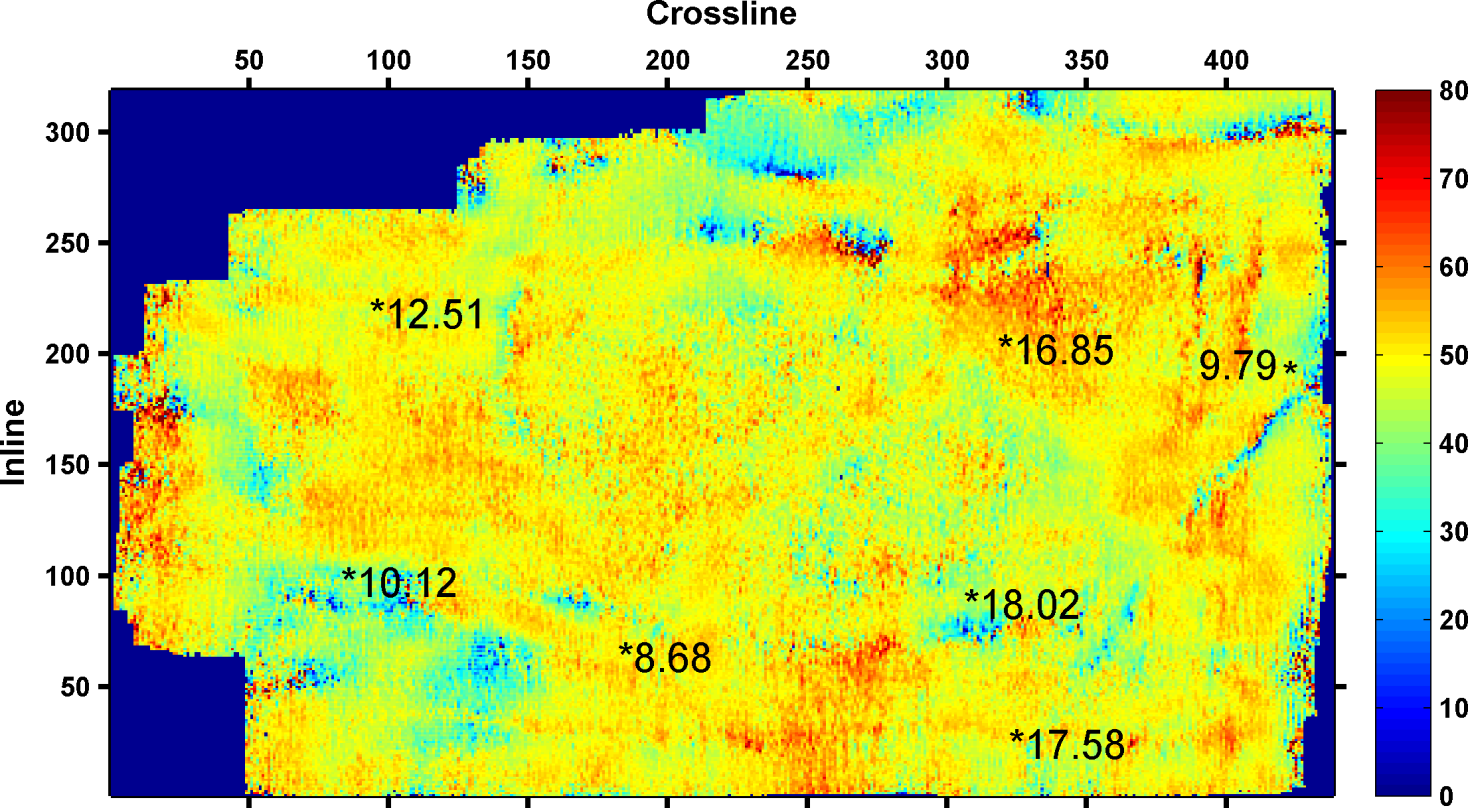
Instantaneous frequency attribute.

**Fig 5:**
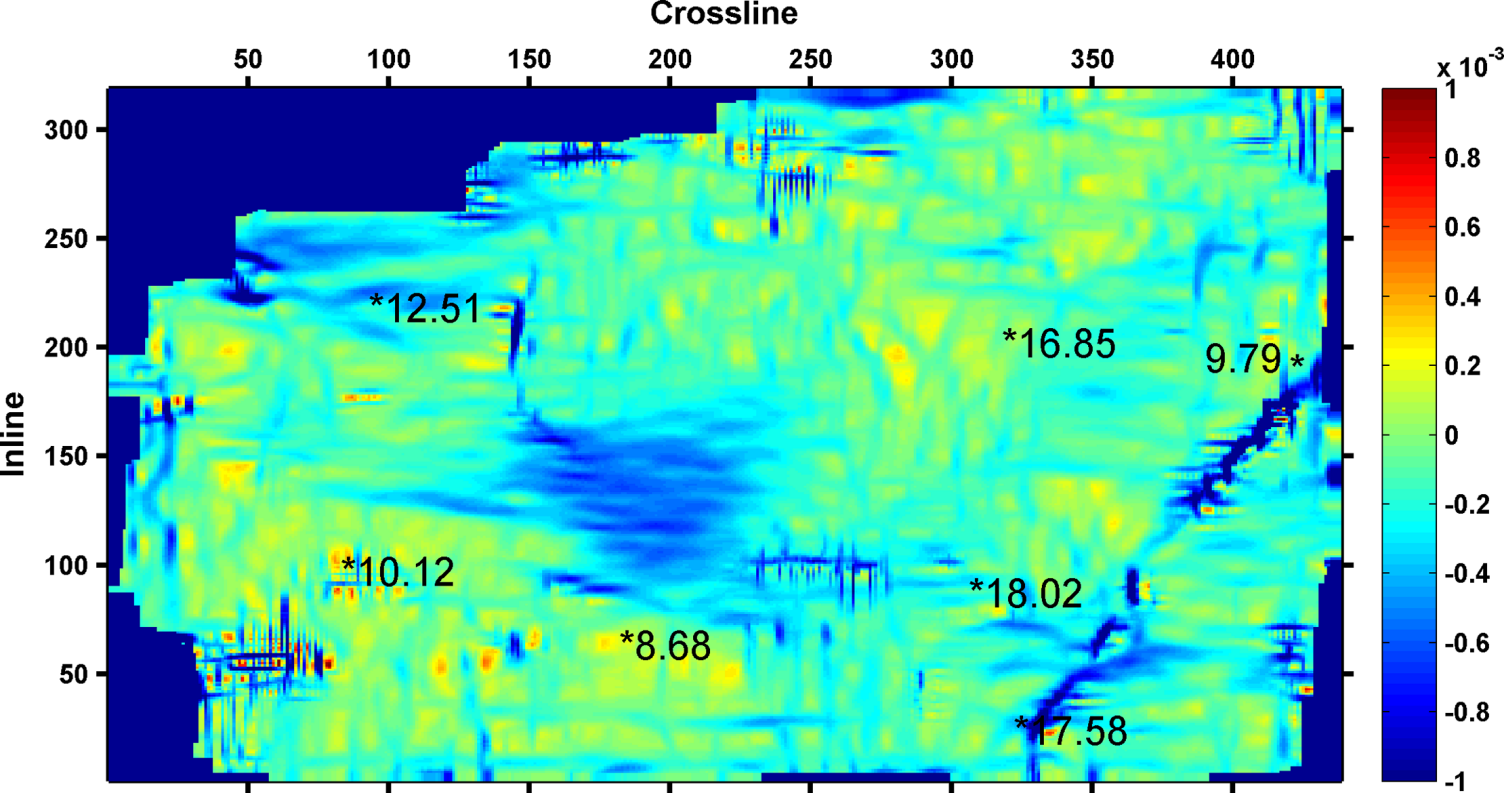
Minimum negative curvature attribute.

As shown in Table 3, the minimum RMSE between the predicted and actual values is 0.6305, when four seismic attributes are selected. Thus, the final seismic attributes selected are as follows: instantaneous amplitude, instantaneous frequency, instantaneous bandwidth, and minimum negative curvature. The four seismic attributes selected along 3coal seam of Qinshui Basin is shown in Figs 2–5. Wells with a high CBM content are located in the strong instantaneous amplitude area, and wells with a low CBM content are located in the weak instantaneous amplitude area.

(The marked values in the figure is content of CBM for wells, the unit is m^3^/t)

As shown in Figs 3–5, the well with a high CBM content is located in the area with a low instantaneous bandwidth, low instantaneous frequency, and high minimum negative curvature, whereas the well with a low CBM content is located in the area with a high instantaneous bandwidth, high instantaneous frequency, and low minimum negative curvature. However, the correlation between the CBM content and these three seismic attributes is not as significant as instantaneous amplitude.

**Fig 6:**
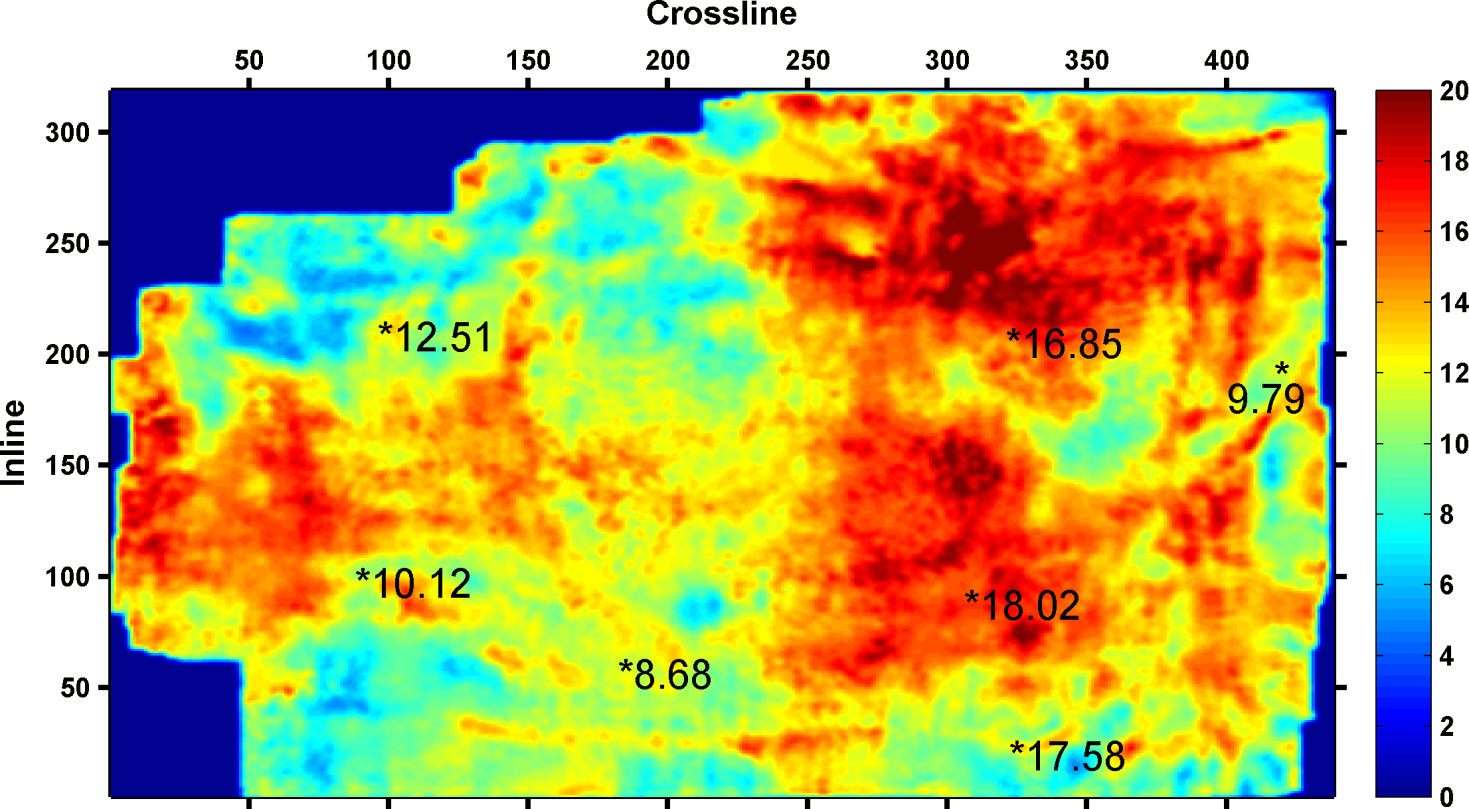
Prediction results of CBM content.

The four types of seismic attributes were used to predict the CBM content of the whole work area based on SVM, and the results are shown in Fig 6.

Fig 6 shows the prediction results of CBM content. In Fig 6, two wells (measured CBM content18.02m^3^/t and 16.85m^3^/t) are located in areas with a high CBM content. Three wells (measured CBM content 10.12m^3^/t, 9.79m^3^/t, and 8.68m^3^/t) are located in areas with the low CBM content. Two wells (measured CBM content 17.58m^3^/t and 12.51m^3^/t) are located in areas with medium CBM content. The prediction results were compared with the measured CBM content, and there is only one well (measured CBM content 17.58m^3^/t) that does not match with the actual value. The prediction results are mainly consistent with the actual values with an accuracy of 85.7%.

**Fig 7:**
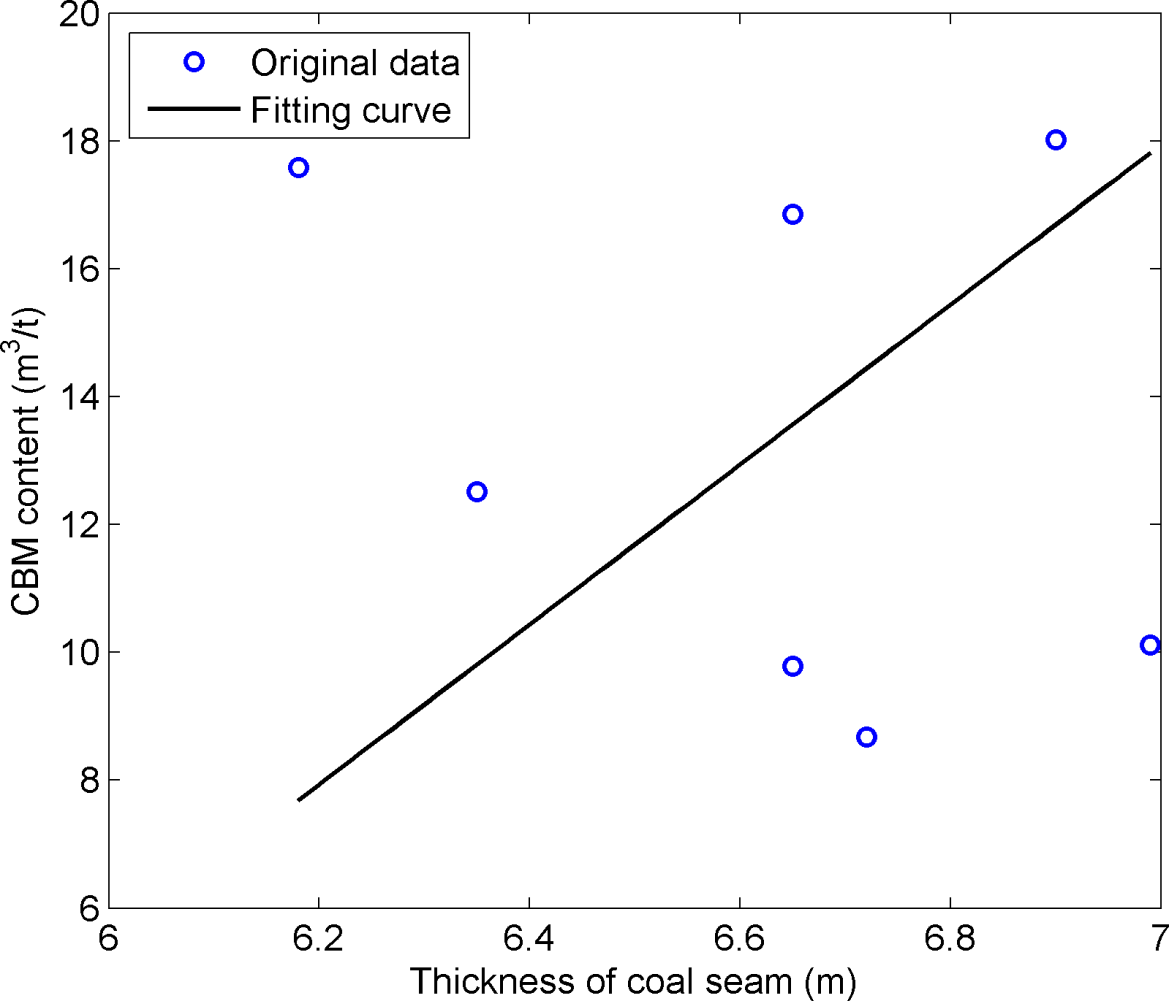
The diagram of coal seam thickness and CBM content.

A certain thickness of coal seam is the base of CBM reservoir. The coal reservoir provides both sources and reservoir spaces for CBM. The thicker the coal seam, the better the production of CBM [27]. Fig 7 shows the relationship between the CBM content and coal seam thickness in this work area. It can be seen from Fig 7 that an area with a larger thickness of coal seam has a higher content of CBM.

**Fig 8:**
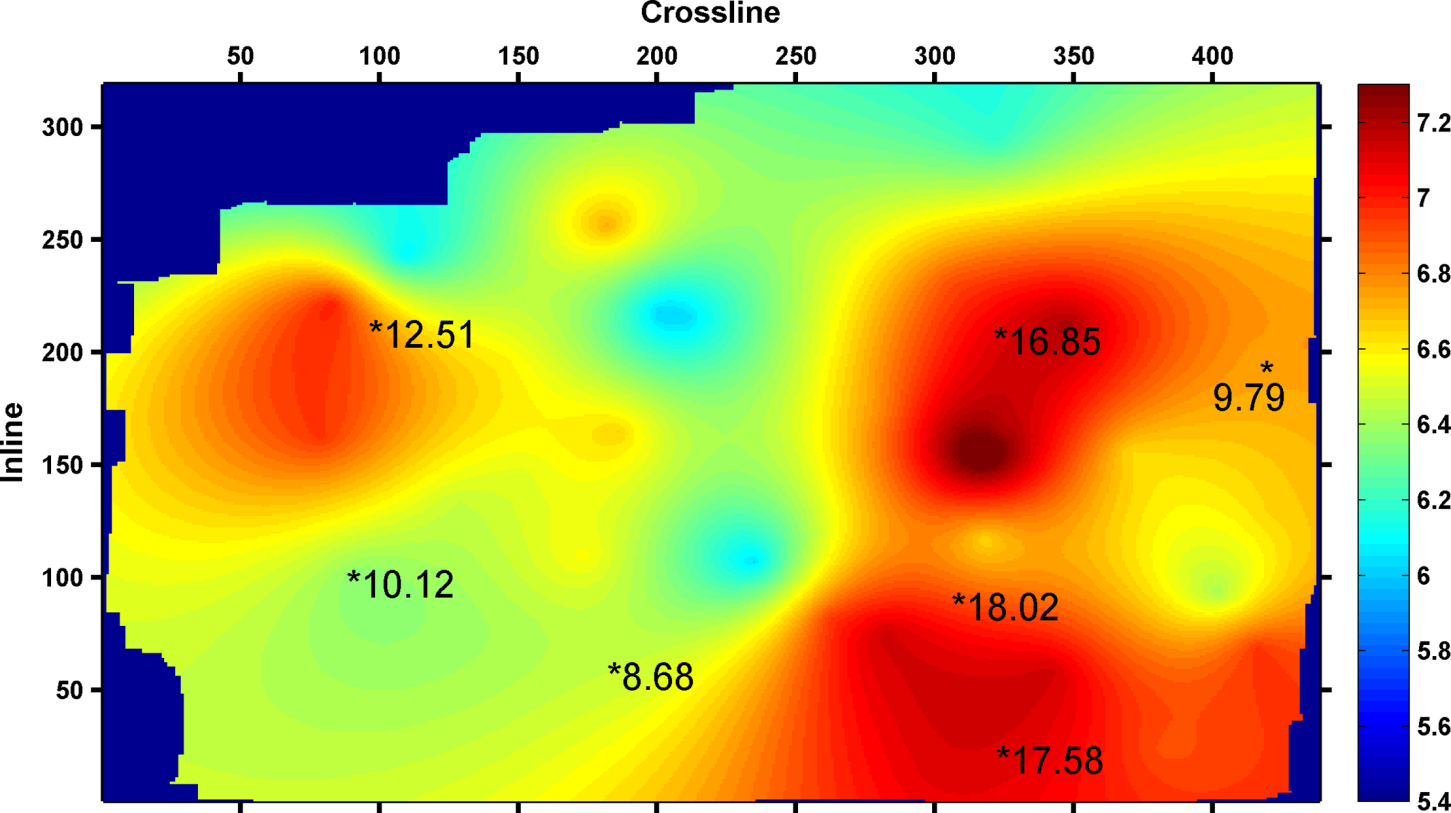
The coal seam thickness distribution.

Fig 8 shows the coal seam thickness distribution in this area. There are three thick coal seam areas nearby the location of wells (measured CBM contents of 16.85m^3^/t, 12.51m^3^/t, and 18.02m^3^/t), as shown in Fig 8.

**Fig 9:**
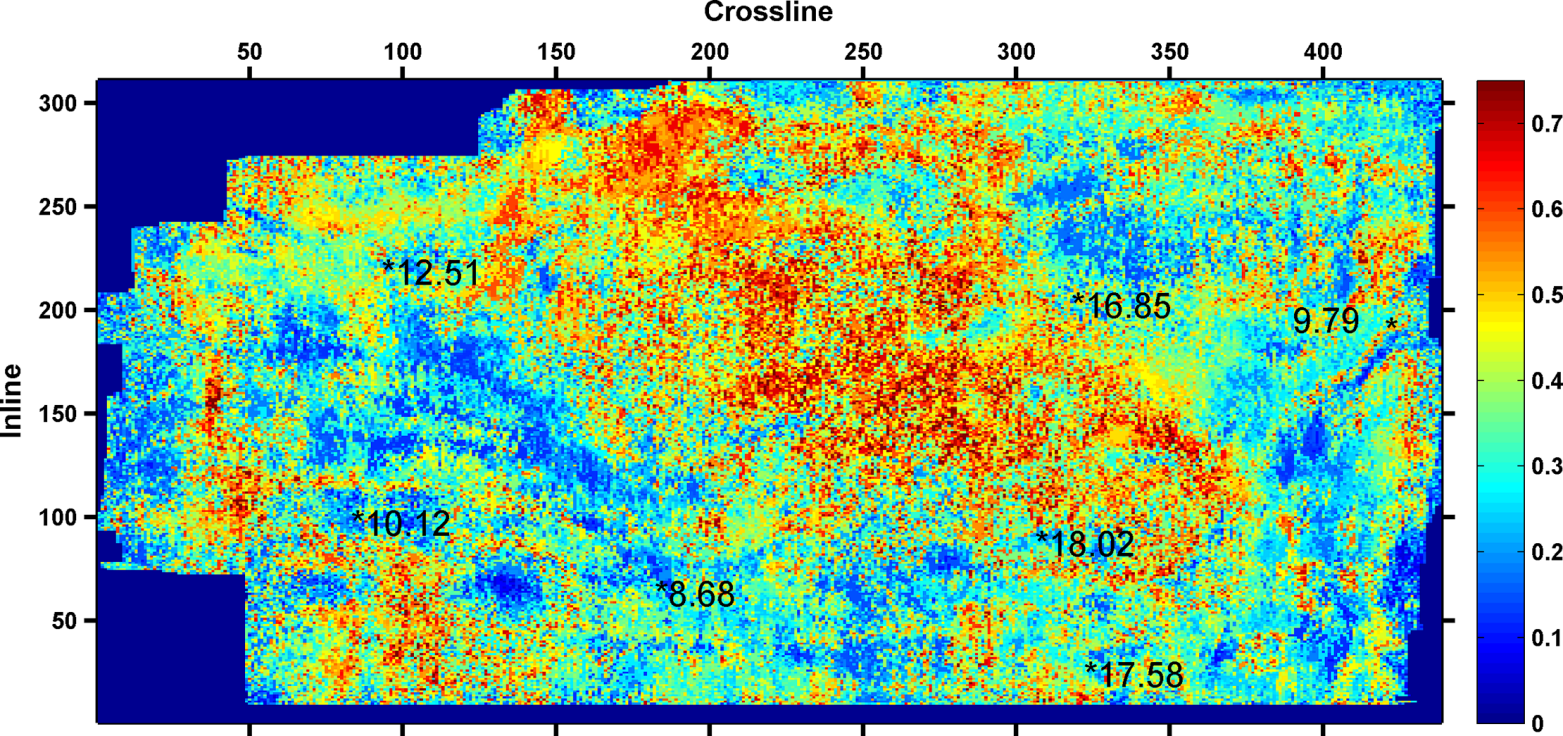
The multiple seismic information fusion results of reflected wave groups in 3coal seam [17].

Fig 9 shows the results of Qi's literature, which represent the multiple seismic information fusion results of 3coal seam. Higher values imply a greater possibility of a high CBM content. The area possible of containing high CBM is between the well with a measured CBM content of 12.51m^3^/t and the well with a measured CBM content of 16.85m^3^/t. The area possible of containing high CBM (Fig 9) is different from the area of the thick coal seam (Fig 8). However, there are three areas with high CBM contents near the location of the wells (measured CBM contents of 16.85m^3^/t, 12.51m^3^/t, and 18.02m^3^/t), as shown in Fig 6. The scope and positions of areas with high CBM content, which were predicted by this study (Fig 6) are essentially consistent with the thickness of the coal seam.

Thus by contrasting and analyzing Fig 6, Fig 8 and Fig 9, the selection method of seismic attributes based on GRD and SVM could effectively select seismic attributes, and the selection results of seismic attributes are more reasonable. The scope and positions of areas with high CBM content, which were predicted by the selection of seismic attributes, are basically consistent with the thickness of the coal seam.

**Fig 10:**
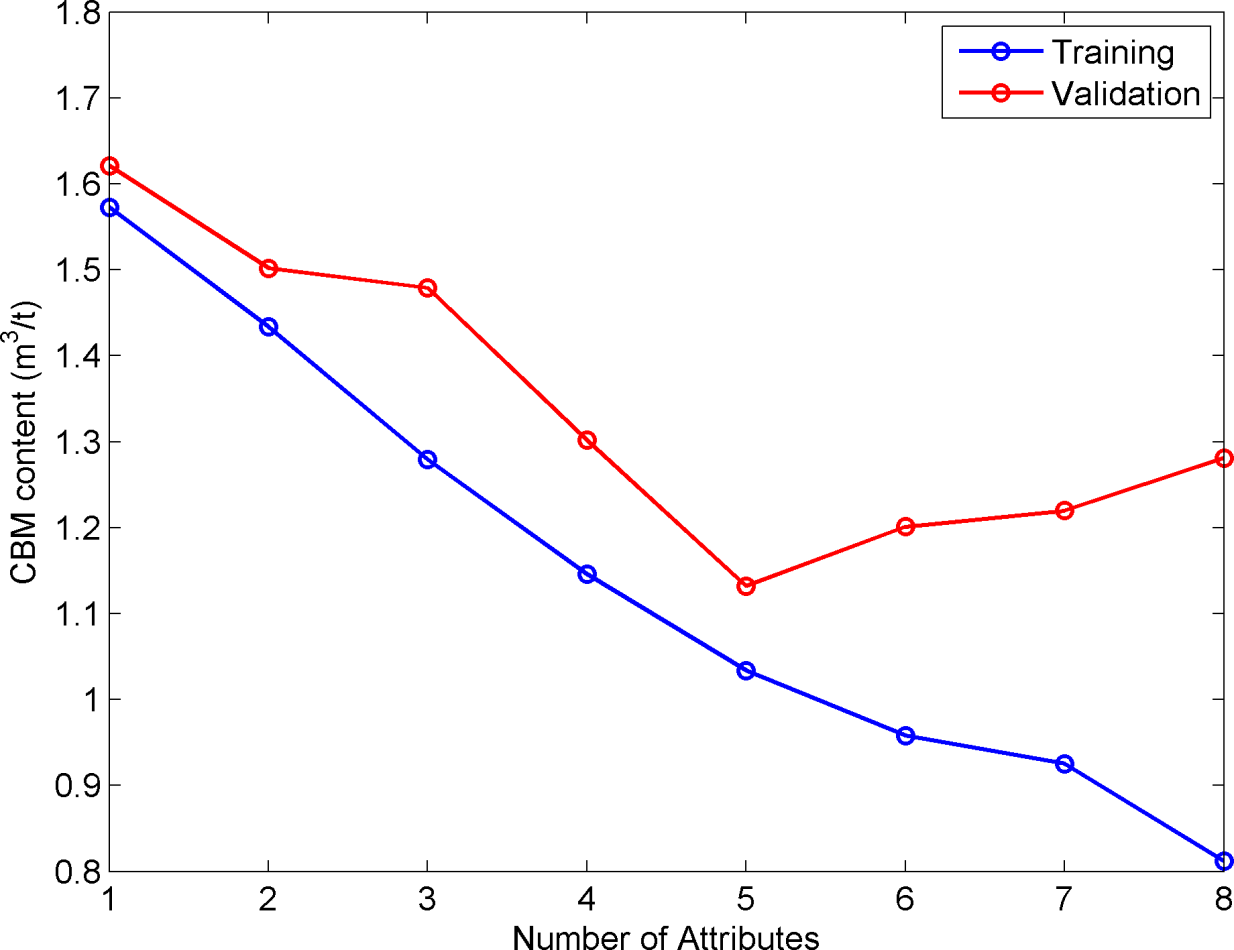
Error analysis result when 8 attributes were used in stepwise regression. Minimum errors occurred when 5 attributes were used.

We also use the 8 seismic attributes: instantaneous amplitude, instantaneous bandwidth, instantaneous main frequency, instantaneous frequency, minimum negative curvature, instantaneous phase, maximum positive curvature, and absorption attribute to carry out Step-wise regression. Fig 10 is a validation plot for the Step-wise regression analysis. The blue (lower) curve shows the error calculated using the Training Data. The red (upper) curve shows the error calculated using the Validation Data. Fig 10 shows that when 5 seismic attributes are selected, the validation error is the smallest. When 6 or more attributes are used, the validation error increases, meaning that these additional attributes are over-fitting the data. The RMSE of Step-wise regression is much larger than the method proposed in this paper. This also illustrates the effectiveness of the method presented in this paper.

## Conclusions

1. The novel method uses GRD to achieve primary selection and applies SVM to obtain the final selection of seismic attributes. This can effectively remove redundant seismic attributes, which can be used for the selection of seismic attributes.
2. Instantaneous amplitude, instantaneous bandwidth, instantaneous frequency, and minimum negative curvature can be used to predict the CBM content. The CBM content and the thickness of the coal seam have a strong corresponding relation.
3. The application of the novel method for predicting the CBM content in southern Qinshui basin, China, illustrates the effectiveness of the seismic attributes selection method presented in this paper.
4. The selection method of seismic attributes based on GRD and SVM requires practical applications in order to verify its validity, and the specific approach would perform multiple blind wells test, which requires a number of wells in the study area.

## Supporting information

S1 File The data is used in “application to seismic data” section. (TXT)

S2 File The data is used in “application to seismic data” section. (TXT)

S3 File The data is used in “application to seismic data” section. (TXT)

S4 File The data is used in “application to seismic data” section. (TXT)

S5 File The data is used in “application to seismic data” section. (MAT)

S6 File The data is used in “application to seismic data” section. (TXT)

S7 File The data is used in “application to seismic data” section. (TXT)

S8 File The data is used in “application to seismic data” section. (MAT)

S9 File The data is used in “application to seismic data” section. (TXT)

## References

[1] Chopra S, Marfurt K. Seismic attributes-A historical perspective. Geophysics.2005, 70, 3SO–28SO. 10.1190/1.2098670.

[2] Fu D, Sullivan E, Marfurt K. Rock-property and seismic attribute analysis of a chert reservoir in the Devonian Thirty-one Formation, west Texas, USA. Geophysics. 2006, 71, B151–B158. 10.1190/1.2335636.

[3] Torrado L, Mann P, Bhattacharya J. Application of seismic attributes and spectral decomposition for reservoir characterization of a complex fluvial system: Case study of the Carbonera Formation, Llanos foreland basin, Colombia. Geophysics. 2014, 79, B221–B230. 10.1190/geo2013-0429.1.

[4] Wang Z, Gao J, Wang D, Wei Q. 3D seismic attributes for a tight gas sand reservoir characterization of the eastern Sulige gas field, Ordos Basin, China. Geophysics. 2015, 80, B35–B43. 10.1190/geo2014-0362.1.

[5] Taner M, Koehler F, Sheriff R. Complex seismic trace analysis. Geophysics. 1979, 44,1041–1063. 10.1190/1.1440994.

[6] Bahorich M, Farmer S. 3-D seismic discontinuity for faults and stratigraphic features: the coherence cube. The Leading Edge.1995, 14, 1053–1058. 10.1190/1.1437077.

[7] Kalkomey C. Potential risks when using seismic attributes as predictors of reservoir properties. The Leading Edge. 1997, 16, 247–251. 10.1190/1.1437610.

[8] Barnes A. Redundant and useless seismic attributes. Geophysics. 2007, 72, P33–P38. 10.1190/1.2716717.

[9] Chopra S, Marfurt K. Seismic attributes for prospect identification and reservoir characterization Society of Exploration Geophysicists and European Association of Geoscientists and Engineers, USA, 2007,1–45.

[10] Dorrington K, Link C. Genetic-algorithm/neural-network approach to seismic attribute selection for well-log prediction. Geophysics. 2004, 69, 212–221. 10.1190/1.1649389.

[11] Fomel S. Local seismic attributes. Geophysics. 2007, 72, A29–A33. 10.1190/1.2437573.

[12] Ahmed O, Abdel-Aal R, AlMustafa H. Reservoir property prediction using abductive networks. Geophysics.2010, 75, P1–P9. 10.1190/1.3298443.

[13] Hampson D, Schuelke J, Quirein J. Use of multi-attribute transforms to predict log properties from seismic data. Geophysics. 2001, 66, 220–236. 10.1190/1.1444899

[14] Gao J, Wang J, Yun M, Huang B, Zhang G. Seismic attributes optimization and application in reservoir prediction. Applied Geophysics. 2006, 3, 243–247. 10.1007/s11770-006-4007-z.

[15] Zhang C, Lu W. Seismic attributes selection based on SVM for hydrocarbon reservoir prediction. SEG Denver 2010 Annual Meeting, 2010, 1586–1590.

[16] Iturraran-Viveros U. Smooth regression to estimate effective porosity using seismic attributes. Journal of Applied Geophysics. 2012, 76, 1–12. 10.1016/j.jappgeo.2011.10.012.

[17] Qi X, Zhang S. Application of seismic multi-attribute fusion method based on D-S evidence theory in prediction of CBM-enriched area. Applied Geophysics. 2012, 9, 80–86. 10.1007/s11770-012-0317-5.

[18] Gholami A, Ansari H. Estimation of porosity from seismic attributes using a committee model with bat-inspired optimization algorithm. Journal of Petroleum Science and Engineering, 2017, 152, 238–249. 10.1190/tle36030239.1.

[19] Galvis I, Villa Y, Duarte C, Sierra D, Agudelo W. Seismic attribute selection and clustering to detect and classify surface waves in multi-component seismic data by using k-means algorithm. The Leading Edge, 2017, 33(6), 239–248. 10.1190/tle36030239.1.

[20] Li Z, Jiang Y, Guo Q, et al Multi-dimensional variational mode decomposition for bearing-crack detection in wind turbines with large driving-speed variations. Renewable Energy. 2018, 116, 55–73. 10.1016/j.renene.2016.12.013

[21] Jiang Y, Li Z, Zhang C, et al On the bi-dimensional variational decomposition applied to nonstationary vibration signals for rolling bearing crack detection in coal cutters. Measurement Science and Technology, 2016, 27(6), 065103 10.1088/0957-0233/27/6/065103

[22] Li Z, Wu D, Hu C, Terpenny J. An ensemble learning-based prognostic approach with degradation-dependent weights for remaining useful life prediction. Reliability Engineering & System Safety, 2018 10.1016/j.ress.2017.12.016

[23] Deng J. The primary methods of grey system theory Huazhong University Press, Wuhan, 2004.

[24] Vapnik V. The Nature of Statistical Learning Theory. New York: Springer-Verlag, 1995.

[25] Chen X, Huo Q, Lin J, Hu C, Wang Y, Zhao Q, et al The relation between CBM content and the elastic parameters of CBM reservoirs: Reasoning and initial probing. Chinese J Geophys-CH.2013, 56, 2837–2848.

[26] Chen X, Huo Q, Lin J, Wang Y, Sun F, Li W, et al The inverse correlations between methane content and elastic parameters of coal-bed methane reservoirs. Geophysics. 2013, 78, D237–D348, 10.1190/geo2012-0352.1.

[27] Fu X, Qin Y, Wei C. Coalbed Gas Geology, Xuzhou, China University of Mining and Technology Press, 2006.

